# Influence of Canonical and Non-Canonical IFNLR1 Isoform Expression on Interferon Lambda Signaling

**DOI:** 10.3390/v15030632

**Published:** 2023-02-25

**Authors:** John Grayson Evans, Laura A. Novotny, Eric G. Meissner

**Affiliations:** 1 Division of Infectious Diseases, Department of Medicine, Medical University of South Carolina, 135 Rutledge Ave., MSC752, Charleston, SC 29425, USA; 2 Department of Microbiology and Immunology, Medical University of South Carolina, Charleston, SC 29425, USA

**Keywords:** interferons, interferon lambda receptor 1, innate immunity, antiviral, inflammation

## Abstract

Interferon lambdas (IFNLs) are innate immune cytokines that induce antiviral cellular responses by signaling through a heterodimer composed of IL10RB and the interferon lambda receptor 1 (IFNLR1). Multiple *IFNLR1* transcriptional variants are expressed in vivo and are predicted to encode distinct protein isoforms whose function is not fully established. *IFNLR1* isoform 1 has the highest relative transcriptional expression and encodes the full-length functional form that supports canonical IFNL signaling. *IFNLR1* isoforms 2 and 3 have lower relative expression and are predicted to encode signaling-defective proteins. To gain insight into IFNLR1 function and regulation, we explored how altering relative expression of IFNLR1 isoforms influenced the cellular response to IFNLs. To achieve this, we generated and functionally characterized stable HEK293T clones expressing doxycycline-inducible FLAG-tagged IFNLR1 isoforms. Minimal FLAG-IFNLR1 isoform 1 overexpression markedly increased IFNL3-dependent expression of antiviral and pro-inflammatory genes, a phenotype that could not be further augmented by expressing higher levels of FLAG-IFNLR1 isoform 1. Expression of low levels of FLAG-IFNLR1 isoform 2 led to partial induction of antiviral genes, but not pro-inflammatory genes, after IFNL3 treatment, a phenotype that was largely abrogated at higher FLAG-IFNLR1 isoform 2 expression levels. Expression of FLAG-IFNLR1 isoform 3 partially augmented antiviral gene expression after IFNL3 treatment. In addition, FLAG-IFNLR1 isoform 1 significantly reduced cellular sensitivity to the type-I IFN IFNA2 when overexpressed. These results identify a unique influence of canonical and non-canonical IFNLR1 isoforms on mediating the cellular response to interferons and provide insight into possible pathway regulation in vivo.

## 1. Introduction

Type-I (alpha and beta; IFNA/IFNB) interferons (IFNs) and lambda IFNs (IFNL) are innate immune cytokines produced upon cellular detection of viral infection [1]. After secretion, IFNs signal in autocrine and paracrine fashion to induce interferon-stimulated genes (ISGs), many of which have antiviral properties, through JAK-STAT-mediated signaling [2,3,4,5]. Type-I IFNs have activity on most cell types and tissues, as the type-I IFN receptor complex IFNAR1/IFNAR2 is expressed by all nucleated cells [6]. Type-I IFNs have been formulated for therapeutic use, for example to treat hepatitis C virus (HCV) and hepatitis B virus (HBV) [7,8], but side effects associated with systemic immune activation, alternative therapeutic options, and inadequate clinical efficacy limit their use [9]. In contrast to IFNAR1/IFNAR2, the interferon lambda receptor 1 (IFNLR1) has restricted expression primarily on cells of epithelial origin located at mucosal sites and anatomical barriers, including the skin, respiratory, gastrointestinal, and reproductive tracts [10,11,12,13,14]. As such, IFNLs are a primary driver of the innate immune response in the initial cells and tissues that are exposed to viral pathogens [15]. IFNLs induce antiviral responses that are less inflammatory than type-I IFNs, likely in part due to restricted IFNLR1 expression, and have been explored as a therapeutic alternative to type-I IFNs [16,17]. Despite demonstrating antiviral activity against multiple viral pathogens including HCV, HBV, and SARS-CoV-2 [8,18,19], IFNLs are not an approved therapy for any infection as of yet. Improved understanding of the regulatory mechanisms underlying the cellular response to IFNLs could provide new insight into how to effectively target this pathway for therapeutic benefit.

Regulation of IFNL signaling, in particular IFNLR1, is incompletely understood as IFNLR1 expression is low in vivo and reagents to detect endogenous IFNLR1 are limiting [20,21]. Transcriptional splice variants predicted to encode distinct IFNLR1 protein isoforms were identified in multiple cell lines and tissues by RNA sequencing [10,11,22], suggesting a potential role for these isoforms in regulation of the IFNL response. However, expression of the corresponding protein isoforms has not been demonstrated. Canonical IFNLR1, produced from transcriptional isoform 1, is signaling-competent and supports JAK-STAT signaling induced by IFNLs [10,11]. In contrast, non-canonical *IFNLR1* isoform 2 and isoform 3 are missing exons that encode key signaling and transmembrane domains, respectively, and are predicted to encode signaling-defective proteins [10,11,22]. Prior studies that evaluated IFNLR1 isoform 1 showed that its overexpression augments the magnitude of IFNL-induced ISG expression without altering the temporal kinetics of gene expression [20,23,24]. In addition, IFNLR1 isoform 1 overexpression broadens the diversity of IFNL-induced genes by increasing formation of STAT1 homodimers that result in expression of IRF1, a pro-inflammatory transcription factor traditionally associated with type-I but not IFNL signaling [20,25].

Much less is known about how and whether non-canonical IFNLR1 isoforms influence pathway regulation. Overexpressed recombinant IFNLR1 isoform 3 has been shown to be secreted, to bind IFNL1, and to negatively regulate ISG induction in HepG2 cells and human peripheral blood mononuclear cells (PBMCs) [26,27]. IFNLR1 isoform 2 expression did not support ISG expression induced by IFNLs in HEK293 cells [22]. Since expression of IFNLR1 is low in vivo and IFNLR1 is less susceptible to negative regulators of IFN signaling than IFNAR1/IFNAR2 [14,28,29], we hypothesized that tight control and relative expression of IFNLR1 isoforms in vivo could play a critical role in pathway regulation.

To explore this hypothesis, we altered relative expression of IFNLR1 isoforms and evaluated the transcriptional cellular response to IFNLs in vitro. First, we asked whether IFNLR1 isoform 1 abundance could be titrated to allow augmentation of select antiviral genes without inducing potentially harmful pro-inflammatory cytokines. Second, we examined whether non-canonical IFNLR1 isoforms 2 and 3 act as negative regulators of the IFNL response in a concentration-dependent manner. We utilized HEK293T cells, previously shown to be a good model for study of IFNLR1 function [22,24], to generate stable clones with doxycycline-inducible expression of each FLAG-tagged IFNLR1 isoform to facilitate receptor visualization and allow precise control of expression.

## 2. Materials and Methods

### 2.1. Generation of Expression Constructs and Stable Cell Lines

Thermo Fisher Scientific’s GeneArt system was used to design FLAG-tagged IFNLR1 constructs. Full cDNA FASTA sequences of IFNLR1 isoforms were retrieved from the NCBI gene database, with IFNLR1 isoform 1 denoting the full-length signaling-capable receptor, isoform 2 missing a portion of the cytoplasmic domain, and isoform 3 missing the transmembrane domain [22,26,27]. A Kozak sequence (ACCAUGG) was added to the extreme 5**′** end to promote ribosomal binding and translation in mammalian cells [30]. The UniProt database was used to design placement of an N-terminal 3X-FLAG tag immediately downstream of the predicted signal sequence (Supplementary Figure S1) [31]. Sequences were codon-optimized to maximize expression in mammalian cells. Each gene construct (henceforth referred to as FLAG-Iso1, FLAG-Iso2, and FLAG-Iso3) was flanked with 5**′** MluI and 3**′** EcoRV restriction enzyme sites to facilitate downstream sub-cloning into the pTRE-Tight-IP doxycycline-inducible expression vector (gift from Dr. Stephen Duncan) [32]. Plasmids were transformed into 5-alpha Competent *E. coli* (C2987H, New England BioLabs, Ipswich, MA, USA) and ampicillin selected (100 µg/mL, A5354-10ML, Sigma Aldrich, St. Louis, MO, USA) followed by purification using an endotoxin-free plasmid maxiprep kit (12362, Qiagen, Hilden, Germany). Nucleotide sequencing (Eurofin Genomics, Louisville, KY, USA) was performed to confirm construct sequence and alignment after cloning.

HEK293T cells were cultured in Dulbecco’s modified Eagle’s medium with F12 (DMEM/F12) supplemented with 10% heat-inactivated fetal bovine serum (FBS) and 1% penicillin/streptomycin, according to ATCC guidelines. Wild-type HEK293T cells were transfected using ViaFect (E4981, Promega, Madison, WI, USA) with empty vector (pTRE-Tight-IP) or pTRE-Tight-IP vectors expressing FLAG-Isoforms and cultured in 3 µg puromycin/mL to generate stable cell lines (A11138-03, Thermo Fisher Scientific, Waltham, MA, USA). Surviving colonies were picked via application of cloning rings, expanded, and continuously cultured in 1 µg puromycin/mL.

### 2.2. Western Blot

To induce construct expression, cells were treated with doxycycline (dox, D9891, Sigma Aldrich) for 24 h prior to interferon treatment. IFNL3 (5259-IL-025/CF, R&D Systems) and IFNA2 (10984-IF, R&D Systems, Bio-Techne, Minneapolis, MN, USA) stock solutions were aliquoted and stored at −80 °C prior to thawing on ice immediately prior to use.

To evaluate construct expression, cellular protein was harvested using RIPA buffer (89900, Thermo Fisher Scientific) supplemented with protease and phosphatase inhibitors (A32959, Thermo Fisher Scientific). Protein concentration was standardized by BCA assay (23227, Thermo Fisher Scientific) prior to electrophoresis using gradient SDS/PAGE gels (Bio-Rad, Hercules, CA, USA).

To detect the FLAG epitope, blots were blocked with 5% milk (M17200-1000.0, Research Products International, Mount Prospect, IL, USA) in TBST (J77500-K8, Thermo Fisher Scientific) for 1 h at room temperature and then incubated with mouse monoclonal anti-FLAG M2 antibody (1:1000, F3165, Sigma-Aldrich) overnight at 4 °C in 1% milk/TBST. After washing, blots were incubated with goat anti-mouse IgG conjugated to HRP (NB7539, Novus Biologicals, Bio-Techne, Minneapolis, MN, USA) for 1 h at room temperature and then developed using ECL (32106, Thermo Fisher Scientific). To detect phosphorylated STAT1 protein (pSTAT1-Y701), blots were blocked in 3% bovine serum albumin (BSA) in TBST for 1 h at room temperature. Blots were then incubated with primary antibody (1:500, mouse monoclonal anti-pSTAT1 (A-2), sc-8394, Santa Cruz Biotechnology, Dallas, TX, USA) overnight at 4 °C in 1% BSA. After washing, blots were incubated with m-IgGk BP-HRP (sc-516102, Santa Cruz Biotechnology) for 1 h at room temperature and developed using ECL. GAPDH was assessed as a loading control by staining with rabbit anti-GAPDH (1:2000, BS2188R, Bioss, Woburn, MA, USA) for 1 h at room temperature, followed by incubation with donkey anti-rabbit IgG conjugated to HRP (1:2000, 406401, BioLegend, San Diego, CA, USA) for 1 h at room temperature. Imaging was performed using FluorChem R (ProteinSimple, Bio-Techne, Minneapolis, MN, USA).

To analyze protein secretion, a 4× volume of chilled 100% acetone was added to culture supernatant, followed by brief vortexing and incubation for 1 h at −20 °C. Samples were then centrifuged 10 min at 15,000× *g*. Protein pellets were air dried for 30 min at room temperature and resuspended in an equal volume of RIPA buffer prior to analysis by western blot.

### 2.3. Flow Cytometry

To detect FLAG-IFNLR1 receptor expression on the cell surface, cells were fixed with fresh 4% paraformaldehyde (PFA; 15710, Electron Microscopy Sciences, Hatfield, PA, USA) for 20 min at 4 °C and stained with rat anti-DYKDDDK Alexa Fluor 488 (1:500, 637317, BioLegend) or rat IgG2a Alexa Fluor 488 Kappa isotype control (1:500, 400525, BioLegend) for 45 min at 4 °C. To detect intracellular receptor expression, cells were fixed and permeabilized using BD Cytofix/Cytoperm Fixation/Permeabilization solution kit (BDB554714, BD, Franklin Lakes, NJ, USA) prior to staining.

To detect pSTAT1, cells were harvested, fixed with 4% PFA for 20 min at 4 °C, and permeabilized in chilled 100% methanol (A411-4, Fisher Scientific) for 15 min at 4 °C. Cells were then stained with mouse anti-Stat1-PE (pY701) (5 µL/100 µL sample, 612564, BD Phosflow) prior to flow cytometry. Mock treated and non-permeabilized cells were used as negative controls.

Flow cytometry was performed using the Guava HT8 Incyte System (Luminex, Austin, TX, USA). Live cells were identified using scatter-area (forward and side scatter) and by staining with a viability dye (1:1000, Zombie Green Fixable Viability, 423111 or 1:1000, Zombie Red Fixable Viability, 423109, BioLegend). FlowJo software was used for analysis.

### 2.4. Gene Expression Assays

To assess receptor function using a dual luciferase assay, cells in 96-well plates were co-transfected with a 5:1 ratio of interferon stimulated response element (ISRE)-firefly luciferase (E4141, Promega) and CMV-renilla luciferase plasmids (E2261, Promega) (300 ng total DNA). Cells were incubated 12–18 h post-transfection, then washed and incubated with fresh media containing ±dox for 24 h, followed by IFNL3 treatment for an additional 24 h. Quantitation of luminescence from lysed cells was determined on a microplate reader (FilterMax F3, Molecular Devices, San Jose, CA, USA) after applying DualGlo Stop and Glo reagent (E2920, Promega) according to manufacturer guidelines.

Expression of individual genes was measured by quantitative reverse transcriptase-polymerase chain reaction (qRT-PCR). Whole cell total RNA was isolated using a Qiagen RNeasy kit (74106, Qiagen). Concentration and quality were assessed using Nanodrop and cDNA was synthesized using a high-capacity RNA-to-cDNA kit (4368814, Thermo Fisher Scientific). qRT-PCR was performed on a CFX96 Real-Time System (Bio-Rad) using TaqMan Fast Advanced Master Mix (4444557, Thermo Fisher Scientific) and commercially available TaqMan primer-probe sets specific for individual genes, including *VIPERIN* (*RSAD2*, Hs00369813_m1), *CXCL10* (Hs0017042_m1), and *IL10RB* (Hs00175123_m1), relative to *GAPDH* (Hs02786624_g1). qRT-PCR experiments were conducted with biological and technical duplicates.

Global transcriptional profiling was performed using the NanoString nCounter Human Immunology v2 Panel. Gene counts were normalized to 15 housekeeping genes using nSolver software. Gene counts below the assay’s threshold of detection (20) were assigned a value of 20 to facilitate analysis of differentially expressed genes (DEGs). Normalized gene counts were compared within each cell line between −dox/+dox and −IFNL3/+IFNL3 conditions to identify DEGs exhibiting at least a 2-fold change in expression. Gene count values were log transformed and adjusted by subtracting the background signal of the assay to facilitate graphical display.

### 2.5. Data and Statistical Analysis

Statistical significance was evaluated in Microsoft Excel and GraphPad Prism 9 using two-tailed Student’s *T*-tests with equal variance. Data are displayed as mean ± standard error of the mean (SEM).

## 3. Results

### 3.1. Generation of Doxycycline-Inducible, FLAG-Tagged IFNLR1 Isoform Expression Constructs and Stable Lines

**Figure 1:**
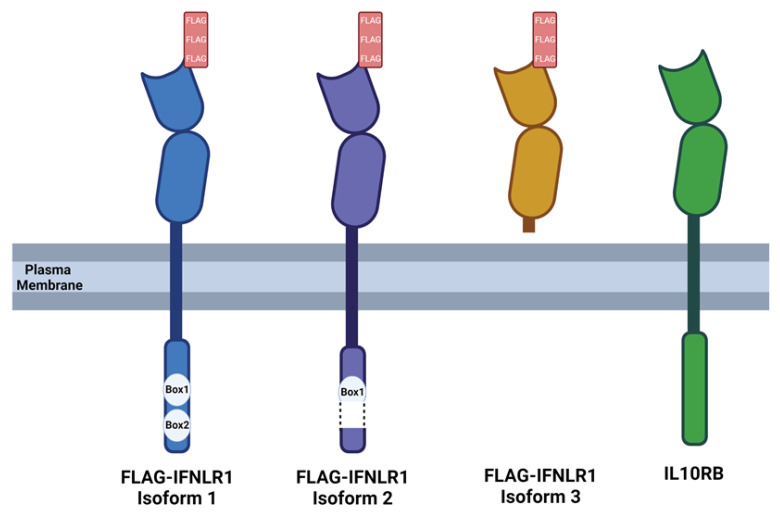
FLAG-IFNLR1 isoforms and co-receptor IL10RB. Schematic depicting the structure of each FLAG-tagged IFNLR1 isoform and IL10RB. Box1 and Box2 depict the Jak1 binding domain that is fully present in FLAG-IFNLR1 isoform 1, truncated in FLAG-IFNLR1 isoform 2, and absent in FLAG-IFNLR1 isoform 3. Image created with BioRender.com using default settings to represent a transmembrane protein.

Endogenous expression of IFNLR1 is low and difficult to detect [20,33]. To facilitate protein visualization and enable precise control of IFNLR1 expression, we synthesized cDNAs that incorporated a 3X-FLAG-epitope at the amino terminus of each IFNLR1 splice variant (referred to as FLAG-Iso1, FLAG-Iso2, FLAG-Iso3) (Figure 1, Supplementary Figure S1) under control of a doxycycline (dox)-inducible promoter [32].

**Figure 2:**
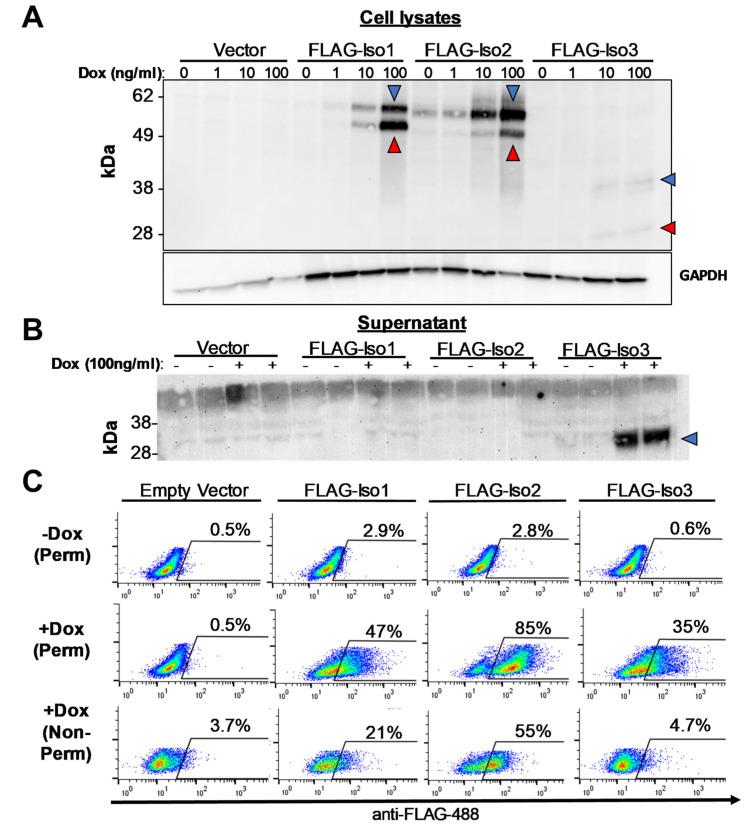
Doxycycline-inducible expression of FLAG-IFNLR1 isoforms in HEK293T cells. (**A**,**B**) Dox-inducible expression of IFNLR1 isoforms in stable HEK293T clone cell lysates (**A**) and culture supernatant (**B**) was detected by western blot using anti-FLAG antibody. Predicted non-glycosylated and glycosylated proteins are denoted by red and blue arrows, respectively. (**C**) Percent of FLAG-positive cells after 24 h induction ±dox (100 ng/mL) followed by permeabilization (perm) or no permeabilization (non-perm), staining with anti-FLAG antibody, and analysis by flow cytometry (representative data, three independent experiments).

We next generated stable HEK293T clones expressing each construct and demonstrated dox-inducible expression of 58 kDa (FLAG-Iso1), 54 kDa (FLAG-Iso2), and 31 kDa (FLAG-Iso3) proteins whose size was consistent with the predicted molecular weight of non-glycosylated protein (Figure 2A). Slightly larger bands were consistent with the predicted molecular weight of glycosylated protein (Figure 2A). Multiple independent clones were evaluated for expression prior to selecting a representative clone for further in-depth study. Several clones had detectable construct expression in the absence of dox, most clearly observed in the FLAG-Iso2 cell line (Figure 2A), even when cells were cultured in media containing dox-free FBS. FLAG-Iso3 had lower relative expression in cell lysates but was readily detected in cellular supernatants (Figure 2B) consistent with it being a secreted protein [27]. Further, while each FLAG-IFNLR1 isoform was detected with intracellular staining, only FLAG-Iso1 and FLAG-Iso2 were detected on the cell surface by flow cytometry (Figure 2C). We observed no impact of FLAG-IFNLR1 isoform expression on endogenous *IL10RB* or *IFNLR1* isoform 1 expression by qRT-PCR and only minimal changes in endogenous expression of *IFNLR1* isoforms 2 and 3 (Supplementary Figure S2). We did not detect any differences in IL10RB surface expression by flow cytometry (FAB874G, R&D Systems) or any differences in cell viability between lines by cell counting or flow cytometry using a Zombie viability stain.

### 3.2. Minimal Expression of FLAG-IFNLR1 Isoform 1 Is Sufficient to Support a Robust Augmentation of the IFNL3 Response

**Figure 3:**
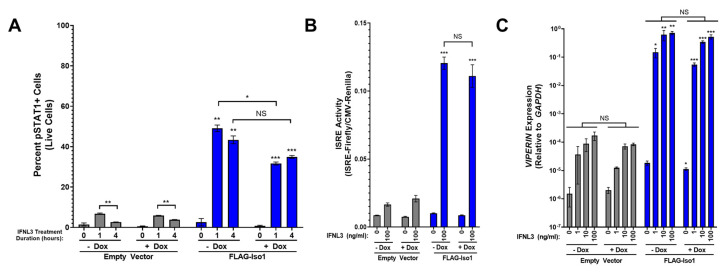
FLAG-Iso1 overexpression augments the cellular response to IFNL3. (**A**) Quantitation of percent pSTAT1+ in HEK293T-EV and FLAG-Iso1 cells after 0, 1, and 4 h of IFNL3 treatment (100 ng/mL) (representative data, two independent experiments). (**B**) HEK293T-EV and FLAG-Iso1 cells were co-transfected with plasmids encoding Firefly Luciferase under control of an ISRE promoter and Renilla Luciferase under control of a CMV promoter. Cells were treated ±dox (100 ng/mL) for 24 h prior to mock or IFNL3 (100 ng/mL) treatment for 24 h and harvested for dual luciferase assay (representative data, three independent experiments). (**C**) HEK293T-EV and FLAG-Iso1 cells were treated ±dox (100 ng/mL) for 24 h prior to IFNL3 treatment for 24 h (0, 1, 10, or 100 ng/mL), then harvested for qRT-PCR analysis of *VIPERIN* and *GAPDH* (representative data, four independent experiments). Statistical significance represented by lone asterisks reflect comparisons between identically treated EV and FLAG-Iso1 cells. Statistical significance represented by bars and asterisks reflect comparisons between -dox and +dox conditions within each cell line. Error bars represent standard error of the mean. * = *p* < 0.05, ** = *p* < 0.01, *** = *p* < 0.001. NS = not significant.

As IFNL binding to IFNLR1/IL10RB results in STAT1 phosphorylation (pSTAT1) [34,35], we examined how relative FLAG-Iso1 expression influenced IFNL3-dependent pSTAT1 using flow cytometry. We observed robust pSTAT1 signal in both −dox and +dox IFNL3-treated FLAG-Iso1 cells relative to HEK293T-empty vector (EV) IFNL3-treated control cells (Figure 3A, representative flow plots in Supplementary Figure S3). Minimal pSTAT1 was detected in mock-treated FLAG-Iso1 cells, indicating expression of FLAG-Iso1 alone did not result in pSTAT1 formation. These data suggest that minimal amounts of FLAG-Iso1 in −dox conditions, below the level that could be detected by flow cytometry or western blot (Figure 2), were sufficient to allow phosphorylation of STAT1 after IFNL3 treatment. We observed reduced pSTAT1 abundance in HEK293T-EV cells between 1 and 4 h of IFNL3 treatment, but no significant decline in pSTAT1 between 1 and 4 h in either −dox or +dox treated FLAG-Iso1 cells (Figure 3A). We also consistently observed lower pSTAT1 in +dox relative to −dox FLAG-Iso1 cells treated with IFNL3, most notably after 1 h of treatment, suggesting excess FLAG-Iso1 expression could partially reduce STAT1 phosphorylation. No significant differences in pSTAT1 were observed at intermediate dox concentrations (1 and 10 ng/mL).

In canonical IFNL signaling, pSTAT1 heterodimerizes with pSTAT2 and associates with IRF9 to form ISGF3, which translocates to the nucleus to bind interferon-stimulated response elements (ISREs) to promote transcription of ISGs [34]. To evaluate the impact of FLAG-Iso1 overexpression on ISRE activity, we optimized a dual luciferase reporter assay that allows quantitation of ISRE promoter activity relative to a control CMV promoter. FLAG-Iso1 cells demonstrated a marked increase in ISRE activity only in the presence of IFNL3 and equally in both −dox and +dox conditions (Figure 3B).

To evaluate how FLAG-Iso1 influences expression of specific ISGs, HEK293T-EV and FLAG-Iso1 cells were dox-induced and IFNL3-treated followed by quantitation of the antiviral ISG *VIPERIN* relative to *GAPDH*. In −dox conditions, FLAG-Iso1 cells exhibited a marked increase in *VIPERIN* expression relative to HEK293T-EV cells (~4000-fold) that was not further augmented in +dox conditions (Figure 3C). Culture of FLAG-Iso1 lines in media supplemented with dox-free FBS did not reduce IFNL3-induced *VIPERIN* expression in −dox conditions. *VIPERIN* was not induced in mock-treated FLAG-Iso1 cells and was minimally induced in HEK293T-EV cells treated with IFNL3, which indicated that *VIPERIN* induction was both IFNL3 and FLAG-Iso1 dependent. No changes in cell viability were observed as a result of dox-induction or IFNL3 treatment.

To further verify the specificity of the observed response, FLAG-Iso1 cells were pre-incubated with anti-IFNLR1 neutralizing antibody prior to IFNL3 treatment. ISRE activity was significantly reduced in −dox conditions, but not in +dox conditions, suggesting that the IFNL response was specifically mediated through IFNLR1 and that antibody inhibition could be overcome with excess amounts of FLAG-Iso1 (Supplementary Figure S4).

Taken together, these data demonstrate that minimal FLAG-Iso1 was sufficient to support a marked augmentation of IFNL3 signaling which could not be further increased by greater receptor expression levels.

### 3.3. Non-Canonical FLAG-IFNLR1 Isoform 2 Differentially Modulates the Cellular Response to IFNL3 Dependent on Relative Receptor Expression

IFNLR1 isoform 2 is predicted to harbor a deletion in a portion of the JAK1 binding domain (Figure 1) [36], and a prior evaluation did not identify capacity for this isoform to support canonical IFNL signaling [22]. We hypothesized that IFNLR1 isoform 2 could function as a dominant negative receptor by binding extracellular ligand without supporting pSTAT1 formation and ISG gene expression, and thus influence the activity of canonical IFNLR1.

**Figure 4:**
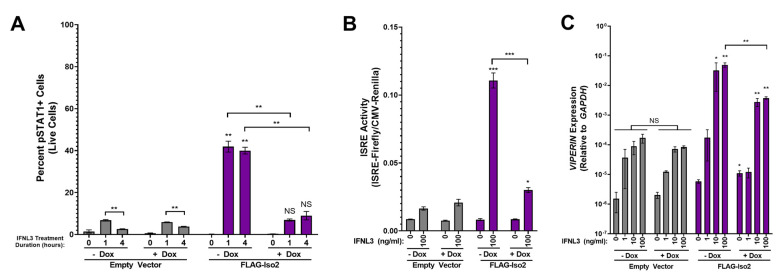
FLAG-Iso2 overexpression differentially influences the cellular response to IFNL3 based on receptor abundance. (**A**) pSTAT1 (two independent experiments), (**B**) ISRE activity (five independent experiments), and (**C**) *VIPERIN* expression (three independent experiments) were analyzed as described in Figure 3. Statistical significance represented by lone asterisks reflect comparisons between identically treated EV and FLAG-Iso2 cells. Statistical significance represented by bars and asterisks reflect comparisons between −dox and +dox conditions within each cell line. Error bars represent standard error of the mean. * = *p* < 0.05, ** = *p* < 0.01, *** = *p* < 0.001. NS = not significant.

To our surprise, pSTAT1 was robustly induced in FLAG-Iso2 cells in −dox, but not +dox conditions, after 1 or 4 h of IFNL3 treatment (Figure 4A, representative flow plots in Supplementary Figure S3). Consistent with this finding, we observed augmented ISRE activity in IFNL3-treated −dox FLAG-Iso2 cells, which was markedly reduced in +dox conditions (Figure 4B). We also observed augmented *VIPERIN* expression in IFNL3-treated −dox FLAG-Iso2 cells that was reduced ~6.5-fold in +dox conditions (Figure 4C). ISRE activity was reduced by pre-incubation of cells with anti-IFNLR1 antibody prior to IFNL3 treatment, demonstrating receptor dependency of the observed phenotype (Supplementary Figure S4). Notably, the augmentation in *VIPERIN* expression in IFNL3-treated −dox FLAG-Iso2 cells was ~14 times lower than that observed in IFNL3-treated -dox FLAG-Iso1 cells.

**Figure 5:**
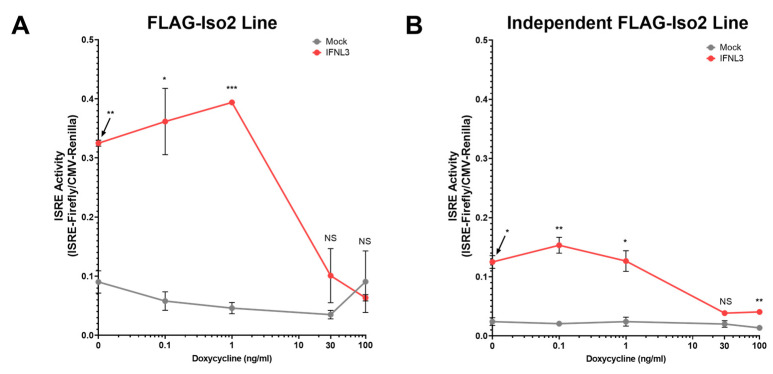
The cellular response to IFNL3 is inversely proportional to FLAG-Iso2 abundance. Two independent FLAG-Iso2 stable lines were transfected with CMV-Renilla and ISRE-Firefly plasmids and dox-treated (dose range between 0–100 ng/mL) for 24 h. Cells were then treated with IFNL3 (100 ng/mL) for 24 h prior to dual luciferase assay. (**A**) FLAG-Iso2 clonal line characterized in Figure 4 and (**B**) an additional independent FLAG-Iso2 clonal line (representative data, two independent experiments). Statistical analysis compares IFNL3 relative to mock-treated cells at a given dox concentration. Error bars represent standard error of the mean. * = *p* < 0.05, ** = *p* < 0.01, *** = *p* < 0.001. NS = not significant.

To further evaluate the dose dependency of this unexpected phenotype, we titrated FLAG-Iso2 expression by varying dox concentration (0–100 ng/mL) prior to IFNL3 treatment (relative expression assessed by flow cytometry and western blot is shown in Supplementary Figure S5). We observed a decline in ISRE activity as dox concentration increased, demonstrating that higher FLAG-Iso2 expression levels reduced the partial augmentation observed at lower receptor expression levels (Figure 5A). We evaluated an independent FLAG-Iso2 clone and found a similar concentration-dependent effect of FLAG-Iso2 expression on ISRE activity (Figure 5B).

Taken together, these data demonstrate that FLAG-Iso2 expression partially augments the IFNL3 response at low receptor expression levels, a phenotype that is attenuated as receptor expression levels are increased by dox-titration.

### 3.4. Overexpression of Non-Canonical IFNLR1 Isoform 3 Partially Augments the Cellular Response to IFNL3

**Figure 6:**
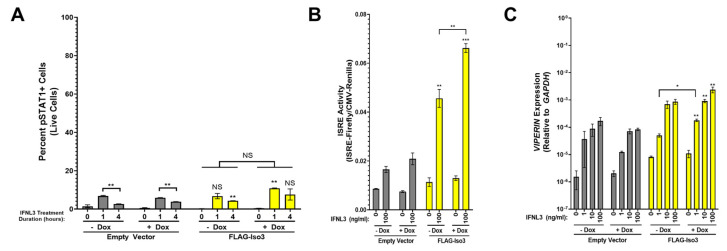
FLAG-Iso3 expression partially augments IFNL signaling. (**A**) pSTAT1 (two independent experiments), (**B**) ISRE activity (three independent experiments), and (**C**) *VIPERIN* expression (three independent experiments) were analyzed as in Figure 3. Statistical significance represented by lone asterisks reflect comparisons between identically treated EV and FLAG-Iso3 cells. Statistical significance represented by bars and asterisks reflect comparisons between −dox and +dox conditions within each cell line. Error bars represent standard error of the mean. * = *p* < 0.05, ** = *p* < 0.01, *** = *p* < 0.001. NS = not significant.

We next evaluated the effect of FLAG-Iso3 overexpression, as IFNLR1 isoform 3 has been shown to inhibit expression of ISGs in IFNL-treated HepG2 cells and PBMCs [26,27]. Contrary to these prior reports, we observed a small but significant increase in pSTAT1 in FLAG-Iso3 cells in +dox conditions after IFNL3 treatment, but not after mock treatment (Figure 6A, representative flow plots in Supplementary Figure S3). FLAG-Iso3 cells also demonstrated a modest increase in ISRE activity in both −dox and +dox conditions and *VIPERIN* expression in +dox conditions after IFNL3 treatment (Figure 6B,C). Notably, the magnitude of IFNL3-induced *VIPERIN* expression (−dox: ~5-fold induction; +dox: ~29-fold induction) was substantially lower than that observed in FLAG-Iso1 and −dox FLAG-Iso2 cells.

### 3.5. Overexpression of FLAG-Iso1 Uniquely Augments Expression of Inflammatory Genes

**Figure 7:**
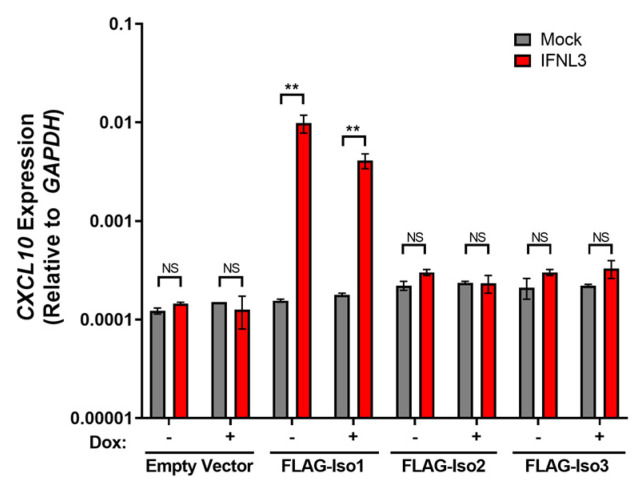
FLAG-Iso1 overexpression uniquely supports expression of the pro-inflammatory gene *CXCL10*. HEK293T stable lines were induced ±dox (100 ng/mL) for 24 h, then mock or IFNL3 (100 ng/mL) treated for an additional 24 h, then evaluated by qRT-PCR for *CXCL10* expression relative to *GAPDH* (representative data, two independent experiments). Error bars represent standard error of the mean. ** = *p* < 0.01, NS = not significant.

Constitutive overexpression of IFNLR1 isoform 1 has been shown to promote de novo expression of the pro-inflammatory cytokine *CXLC10* after IFNL3 treatment [20]. We thus hypothesized that expression levels of FLAG-Iso1, FLAG-Iso2, and FLAG-Iso3 that supported IFNL3-dependent *VIPERIN* expression would also support the capacity of cells to induce *CXCL10*. Consistent with prior studies, we observed augmented *CXCL10* expression in IFNL3-treated FLAG-Iso1 cells in both −dox and +dox conditions (Figure 7). Unexpectedly, expression of FLAG-Iso2 or FLAG-Iso3 did not result in *CXCL10* expression after 24 h of IFNL3 treatment, suggesting non-canonical IFNLR1 isoforms may not support expression of pro-inflammatory genes.

**Figure 8:**
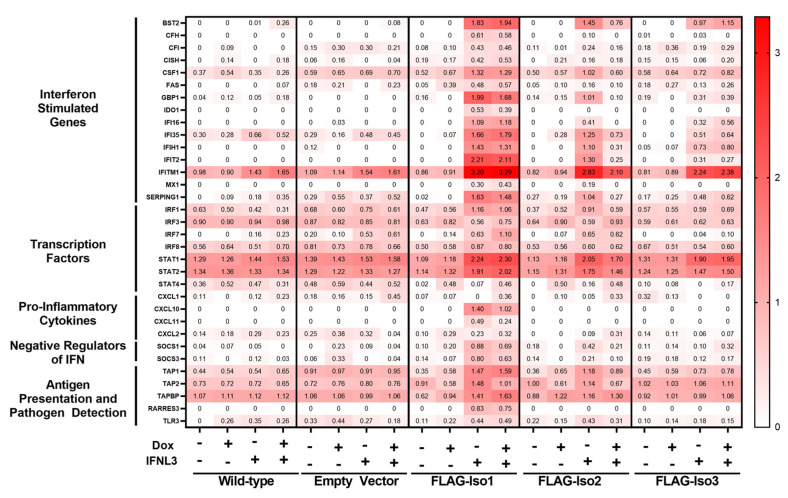
Relative expression of FLAG-IFNLR1 isoforms differentially influences IFNL3-dependent gene expression. HEK293T WT and stable lines were induced ±dox (100 ng/mL) for 24 h prior to treatment ±IFNL3 (100 ng/mL) for an additional 24 h. RNA was collected and gene counts were quantitated using NanoString analysis (nCounter Human Immunology v2 Panel). Data are shown as log transformed normalized counts of differentially expressed genes (>2-fold change in any condition).

To further explore differential induction of antiviral and inflammatory ISGs, we performed gene expression profiling of immune and inflammatory genes using the NanoString Immunology 2.0 panel. We identified 61 differentially expressed genes (DEGs, two-fold change) that demonstrated varied expression upon treatment with dox or IFNL3 (Supplementary Figure S6 and Supplementary File S1). A representative subset of DEGs which include interferon stimulated genes, transcription factors, pro-inflammatory cytokines, negative regulators of IFN signaling, and antigen presentation and pathogen detection genes is displayed in Figure 8.

We identified 40 DEGs when comparing mock and IFNL3-treated FLAG-Iso1 cells, relative to only six and four DEGs affected by IFNL3 in WT and HEK293T-EV lines, respectively, demonstrating that FLAG-Iso1 expression increases the magnitude and breadth of IFNL3-dependent transcriptional activation. Consistent with our prior analysis of *VIPERIN* and *CXCL10* (Figure 3 and Figure 7), IFNL3-treated FLAG-Iso1 cells exhibited upregulation of multiple antiviral ISGs (e.g., *IFITM1*, *IFIT2*, *BST2*) as well as pro-inflammatory markers and chemokines (*IRF1*, *CXCL10*, *CXCL11*), irrespective of dox-induction and consistent with prior reports [20,24,25]. In addition, we observed upregulation of known negative regulators of IFN signaling (*SOCS1*, *SOCS3*) [37,38,39], critical mediators of IFN signaling (*STAT1*, *STAT2*), and genes important for pathogen detection (*RARRES3*, *TLR3*) and antigen presentation (e.g., *TAP1*, *TAP2*).

We identified only 20 DEGs when comparing mock and IFNL3-treated FLAG-Iso2 cells, 18 of which were also identified in the FLAG-Iso1 dataset. Similar to FLAG-Iso1 cells, but to a lesser extent, we observed an IFNL3-dependent augmentation of select ISGs (e.g., *IFITM1*, *STAT1*/*STAT2*) and negative regulators of IFN signaling (*SOCS1*) in −dox conditions, an augmentation that was notably reduced by the addition of dox, consistent with Figure 4 and Figure 5. In contrast to the FLAG-Iso1 dataset, we did not observe induction of pro-inflammatory genes after IFNL3 treatment in FLAG-Iso2 cells, regardless of dox induction.

We identified 11 DEGs when comparing mock and IFNL3-treated FLAG-Iso3 cells. We observed partial augmentation of select antiviral ISGs regardless of dox-induction. Similar to FLAG-Iso2, we observed slight induction of *SOCS1* but no induction of pro-inflammatory genes.

Importantly, we observed no change in expression of type-I IFN receptor subunits (*IFNAR1*, *IFNAR2*, Supplementary Figure S7) or apoptosis/cell stress genes (e.g., *BAX*, *BCL2*, *CASP2*) in any condition. This is consistent with the lack of impact of construct expression and IFNL3 treatment on cellular viability we had previously observed by flow cytometry. 

### 3.6. Overexpression of FLAG-Iso1 Reduces the Impact of Type-I IFN Signaling

The type-I IFN response is highly regulated to temper the potentially deleterious effects of pathway overactivation [37,40], and it is established that there is significant cross-talk between type-I and IFNL signaling pathways [21]. Specifically, it has been demonstrated that stimulation of the IFNL system results in potent inhibition of a subsequent type-I IFN response, mediated in part through upregulation of negative regulators of type-I IFN signaling [28]. Consistent with this observation, our NanoString analysis identified that FLAG-IFNLR1 isoform 1 overexpression enhances IFNL3-mediated upregulation of known negative regulators of IFN signaling, including *SOCS1* and *SOCS3* (Figure 8).

**Figure 9:**
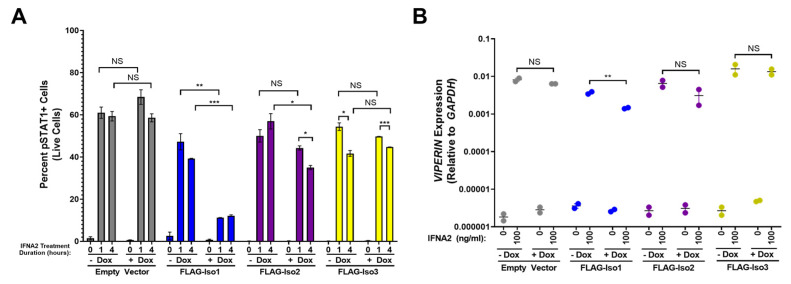
Overexpression of FLAG-Iso1 partially reduces the cellular response to the type-I IFN ligand IFNA2. (**A**) Quantitation of percent pSTAT1+ in HEK293T-EV and FLAG-IFNLR1 isoform cells after 0, 1, and 4 h of IFNA2 treatment (100 ng/mL) (representative data, two independent experiments). (**B**) HEK293T stable lines were ±dox (100 ng/mL) treated for 24 h, then mock or IFNA2 (100 ng/mL) treated for 24 h, then harvested for qRT-PCR analysis of *VIPERIN* and *GAPDH* (representative data, two independent experiments). Statistical significance represented by bars and asterisks reflect comparisons between the indicated conditions. Error bars represent standard error of the mean. * = *p* < 0.05, ** = *p* < 0.01, *** = *p* < 0.001. NS = not significant.

As IFNLR1/IL10RB and IFNAR1/IFNAR2 pathways both utilize several shared signaling molecules, including JAK1 and TYK2, we hypothesized that overexpression of FLAG-IFNLR1 isoforms may also affect the cellular response to type-I IFNs even in the absence of IFNL3 pre-treatment. To test this hypothesis, we evaluated pSTAT1 in HEK293T-EV and FLAG-IFNLR1 isoform lines after stimulation with the type-I IFN IFNA2 (Figure 9A, representative flow plots in Supplementary Figure S3). Relative to HEK293T-EV cells, each FLAG-IFNLR1 isoform line demonstrated a slightly reduced IFNA2-dependent pSTAT1 signal after 1 and 4 h of IFNA2 stimulation (Figure 9A). Surprisingly, we observed a marked reduction in IFNA2-dependent pSTAT1 signal in +dox relative to −dox FLAG-Iso1 cells after IFNA2 treatment. We observed only marginal differences in the IFNA2 response in FLAG-Iso2 and FLAG-Iso3 cells between conditions. To further evaluate this observation, we examined pSTAT1 by western blot after 1 h of treatment with IFNL3 or IFNA2 (Supplementary Figure S8). Consistent with our prior flow cytometry analysis (Figure 3, Figure 4 and Figure 6), the highest pSTAT1 induction after IFNL3 treatment was observed in FLAG-Iso1 cells, irrespective of dox treatment. In contrast, there was a notable reduction in pSTAT1 in IFNA2-treated +dox FLAG-Iso1 cells relative to other lines. As we observed no differences in *IFNAR1* or *IFNAR2* gene expression between lines (Supplementary Figure S7), these data suggest that high levels of FLAG-Iso1 expression may partially inhibit IFNA2-dependent STAT1 phosphorylation.

Next, we evaluated the impact of FLAG-IFNLR1 overexpression on the capacity of IFNA2 to stimulate expression of *VIPERIN*. In HEK293T-EV, FLAG-Iso2, and FLAG-Iso3 cells, we observed robust induction of *VIPERIN* after IFNA2 treatment that was unaffected by pre-treatment with dox (Figure 9B). In contrast, dox-induction of FLAG-Iso1 prior to IFNA2 treatment resulted in a small but significant decrease in *VIPERIN* expression (Figure 9B). Taken together, these results suggest that overexpression of FLAG-Iso1 partially impairs the cellular response to IFNA2.

## 4. Discussion

In this study, we identified a unique influence of relative expression of IFNLR1 isoforms on the cellular transcriptional response to interferons. We demonstrate that minimal overexpression of FLAG-Iso1 is sufficient to markedly augment IFNL3-dependent STAT1 phosphorylation, ISRE-promoter activation, and induction of antiviral and pro-inflammatory genes, a phenotype which could not be further augmented at greater FLAG-Iso1 expression levels. Furthermore, FLAG-Iso1 overexpression partially impaired signaling induced by IFNA2. Surprisingly, we found that FLAG-Iso2 partially augmented IFNL3-dependent antiviral gene expression and phosphorylation of STAT1 at low receptor expression levels, but this augmentation was markedly reduced at greater receptor expression levels. FLAG-Iso3 expression also modestly increased IFNL3-dependent antiviral gene expression and phosphorylation of STAT1, albeit to a much lower extent than either FLAG-Iso1 or FLAG-Iso2. Strikingly, in contrast to FLAG-Iso1, neither FLAG-Iso2 or FLAG-Iso3 supported expression of pro-inflammatory genes after IFNL3 treatment, nor did they substantially impact the cellular response to IFNA2. These data suggest that relative IFNLR1 isoform expression could influence the balance of antiviral and inflammatory genes induced by interferons.

Our observation that FLAG-Iso1 overexpression robustly augments antiviral gene expression is consistent with previous reports [20,24], suggesting that our findings are not an artifact of the addition of a FLAG tag or of the cellular system. In addition, we observed de novo expression of *CXCL10* and other pro-inflammatory genes upon FLAG-Iso1 overexpression and treatment with IFNL3, also consistent with prior reports [20]. Contrary to our hypothesis, we were unable to titrate FLAG-Iso1 to allow selective upregulation of antiviral ISGs without inducing pro-inflammatory genes, as levels of FLAG-Iso1 undetectable by western blot or flow cytometry in −dox conditions were sufficient to support a maximal response to IFNL3 (Figure 2 and Figure 3). This observation would be consistent with the suggested stochastic or bimodal nature of the IFNL response, which suggests cells undergo an “all or nothing” response when stimulated with IFNs [41,42]. These data also suggest that IFNLR1 isoform 1 abundance could be tightly regulated in vivo as a means to control pathway activity and prevent cells from expressing excessive and potentially deleterious pro-inflammatory genes. As it is well-established that tetracycline-inducible promoters allow some extent of construct expression even in the absence of tetracyclines [43,44], use of alternative gene expression control strategies may prove useful in future studies to further assess whether very low levels of receptor expression allow for a titratable phenotype.

There is a high degree of cross-talk between IFN systems [21]. Specifically, IFNL stimulation has been demonstrated to have a potent negative effect on the type-I IFN response through induction of negative regulators of IFN signaling [28], as we observed in IFNL3-treated FLAG-Iso1 cells. As such, we were surprised to observe that overexpression of FLAG-Iso1, even in the absence of IFNL3 pre-treatment, resulted in a marked reduction in IFNA2-dependent pSTAT1 induction (Figure 9). Notably, we observed a significant albeit less marked reduction in pSTAT1 in +dox FLAG-Iso1 cells after IFNL3 treatment (Figure 3A). We speculate that overexpression of excess levels of FLAG-Iso1 could thus influence the immediate cellular response to IFNs by binding and potentially sequestering signaling molecules required for propagation of the IFN response. The less notable differences observed in ISRE promoter activity and ISG expression when comparing −dox and +dox FLAG-Iso1 cells 24 h after IFN stimulation could reflect adequacy of a partial pSTAT1 response to mediate gene expression or may relate to secondary signaling events that occur during in vitro culture.

We were surprised to find that minimal FLAG-Iso2 overexpression partially augmented IFNL3-induced expression of antiviral ISGs without concurrent induction of pro-inflammatory cytokines and chemokines. While FLAG-Iso2 expression led to partial upregulation of the inflammatory transcription factor *IRF1* after IFNL3 treatment (Figure 8), we did not observe augmentation of genes under its transcription control (e.g., *CXCL10*). Consequently, the magnitude of IRF1 induction as a function of FLAG-Iso2 expression may be insufficient to promote widespread changes in pro-inflammatory genes, or alternatively, FLAG-Iso2 may engage signaling mechanisms distinct from FLAG-Iso1. These results differ from a prior report that suggested IFNLR1 isoform 2 does not support IFNL3 signaling [22]. Consistent with this prior report, where IFNLR1 isoform 2 was constitutively expressed off the pEF2 promoter [22], we found that IFNL3 signaling was markedly reduced at high levels of FLAG-Iso2 expression in +dox conditions. We conclude that IFNLR1 isoform 2 may be capable of augmenting IFNL signaling at low, but not high levels of receptor expression.

It has been shown that overexpression of cell surface receptors can decrease the cellular response to their cognate biological stimuli [45], and that overexpression of a receptor subunit without concordant overexpression of downstream signaling molecules can lead to the formation of receptor complexes capable of binding ligands but unable to transduce signals [46]. As FLAG-Iso1 overexpression augmented the IFNL3 response, regardless of dox-induction, we find it less likely that FLAG-Iso2 overexpression diminished cell sensitivity to IFNL3 by titrating necessary signaling components. Rather, it is possible that FLAG-Iso2 influences signaling through endogenous IFNLR1 isoform 1 or can support signaling in tandem with IL10RB only at low levels. While the mechanisms underlying the effect of FLAG-Iso2 on the cellular response to IFNL3 are not clearly established by this work, our data support the hypothesis that IFNLR1 isoform 2 plays a role in modulating the sensitivity of cells to IFNLs and could thus potentially provide a tunable mechanism of IFNL regulation.

In contrast to our findings in IFNA2-treated FLAG-Iso1 cells, pSTAT1 induction was only modestly reduced in +dox FLAG-Iso2 cells and only after 4 h of IFNA2 treatment. Notably, this reduced response was less marked than the reduced response to IFNL3 observed in +dox FLAG-Iso2 cells (Figure 4 and Figure 9). Similarly, we observed no impact of FLAG-Iso2 overexpression on *VIPERIN* expression in IFNA2 relative to IFNL3-treated cells. Thus, FLAG-Iso1 overexpression has a more immediate impact on pSTAT1 induction after IFNA2 relative to IFNL3 treatment, whereas FLAG-Iso2 overexpression has the opposite impact. While the underlying mechanism by which FLAG-Iso1 and FLAG-Iso2 differentially affect signaling by IFNL3 and IFNA2 is not clearly established by this work, we hypothesize that structural differences between FLAG-Iso1 and FLAG-Iso2, with the latter missing a significant portion of its predicted JAK1 binding domain, may contribute. Future work to examine relative differences in recruiting and/or binding of JAK1 and STAT1 to FLAG-Iso1 and FLAG-Iso2 after treatment with interferons will help further elucidate a potential mechanism for these observations.

Overexpression of FLAG-Iso3 resulted in a subtle yet consistent augmentation of IFNL3-dependent STAT1 phosphorylation, ISRE-promoter activation, and induction of the antiviral gene *VIPERIN*, albeit to a much lower extent than FLAG-Iso1 or FLAG-Iso2 (Figure 6). Similar to FLAG-Iso2, FLAG-Iso3 did not support induction of *CXCL10*, and NanoString analysis confirmed that FLAG-Iso3 partially augmented IFNL3-induced antiviral genes but not pro-inflammatory genes (Figure 7 and Figure 8). Prior studies investigating the influence of IFNLR1 isoform 3 on the IFNL response have shown that it can act as an inhibitor of IFNL signaling [26,27]. In HepG2 cells, IFNLR1 isoform 3, also referred to as short IFNLR1, is secreted, can bind IFNL1, and negatively impacts IFNL1-dependent expression of MHCI [27]. In PBMCs, recombinant IFNLR1 isoform 3 binds to the cell surface and increases binding of ligands, but negatively regulates ISG induction [26]. In contrast to our work, these studies utilized recombinant, purified IFNLR1 isoform 3, which differs in concentration and absence of factors present in cellular supernatants, both of which could have influenced the observed differences. Consequently, we speculate that the impact of IFNLR1 isoform 3 may be both receptor concentration- and cell type-dependent, similar to what has been observed for the soluble IFNAR2 isoform [47,48].

Our study has several important limitations. While the use of HEK293T cells for characterization of FLAG-IFNLR1 isoforms was informed by prior published work [22,24], wild type HEK293T cells are largely unresponsive to IFNLs and are deficient in certain aspects of IFN signaling [49,50,51]. In addition, our cell model relies on protein overexpression, which results in supra-physiologic levels of receptor. Consequently, these findings will benefit from further evaluation in cell types and tissues where IFNL signaling is physiologically active, and ideally, through precise manipulation of endogenous IFNLR1 isoform expression. While the focus of this study was to characterize the impact of IFNLR1 isoform overexpression on the cellular transcriptional responses to IFNs, future efforts examining resultant protein expression and susceptibility of modulated cells to viral infection will be necessary to better understand how each isoform contributes to the cellular antiviral response.

Taken together, our data suggest that IFNLR1 isoforms could potentially modulate IFNL signaling by altering both the magnitude of antiviral gene expression as well as mediating differential expression of pro-inflammatory genes. In addition, we demonstrate that overexpression of FLAG-Iso1 and FLAG-Iso2 differentially influence both IFNL and type-I IFN signaling. Improved knowledge of IFNLR1 regulation, including establishing whether differential expression of IFNLR1 isoforms may influence pathway activity in vivo, could help guide future attempts to use IFNLs for therapeutic benefit.

## 5. Conclusions

In conclusion, our study demonstrates that relative expression of each IFNLR1 isoform uniquely influences the magnitude and inflammatory nature of the cellular transcriptional response to interferon treatment. These findings suggest that differential expression of IFNLR1 isoforms in vivo could potentially influence the cellular response to both endogenous and therapeutically administered IFNLs.

## Data Availability

The data presented in this study are available on request from the corresponding author.

## Supplementary Materials

The following supporting information can be downloaded at: https://www.mdpi.com/article/10.3390/v15030632/s1, Supplementary Figure S1: Protein and nucleotide sequences of IFNLR1 isoforms. Unaltered protein sequences downloaded from UniProt database (left column), codon-optimized nucleotide sequences used for Gene Art synthesis (middle column), and FLAG-tagged IFNLR1 isoform protein sequences (right column). Signal sequence is denoted in blue; 3x-FLAG tag is denoted in red; the transmembrane domain is denoted in orange. Supplementary Figure S2: Endogenous IL10RB and IFNLR1 isoform expression in HEK293T stable lines. Cellular gene expression was quantitated by qRT-PCR. Statistical significance represented by asterisks reflect comparisons between EV and FLAG-IFNLR1 isoform cells. Error bars represent standard error of the mean. * = *p* <0.05, ** = *p* < 0.01. NS = not significant. Supplementary Figure S3: Representative flow cytometry plots used for quantification in Figure 3, Figure 4, Figure 6 and Figure 9. Supplementary Figure S4: Effect of pre-incubation with anti-IFNLR1 antibody on the IFNL3 response in FLAG-Iso1 and FLAGIso2 lines. IFNLR1 FLAG-Iso1 (A) and FLAG-Iso2 (B) cells were treated +/− dox (100 ng/mL) for 24 hrs prior to incubation with anti-IFNLR1 antibody or isotype control (anti-IL-28RA antibody, control IgG, R&D Systems, 4 ug/mL) for 1.5 hrs, after which the cells were stimulated with IFNL3 (100 ng/mL) for 24 hrs and harvested for dual luciferase assay (representative data, 2 independent experiments). Error bars represent standard error of the mean. * = *p* < 0.05. NS = not significant. Supplementary Figure S5: Dox-titratable FLAG-Isoform 2 expression in independent HEK293T stable lines. (A) HEK293T-EV, the FLAG-Iso2 line characterized in Figure 4, and an independent FLAG-Iso2 line were treated +/- dox (0, 0.1, 1, 10, or 100 ng/mL) for 24 hr prior to analysis of FLAG expression by flow cytometry. (B) FLAG-Iso2 expression in each line after treatment +/- dox (0, 1, 10, 100 ng/mL) for 24 hr is shown by western blot of cell lysates using anti-FLAG antibody. Supplementary Figure S6: Differentially expressed genes among HEK293T cell lines. HEK293T WT and stable lines were induced +/- dox (100 ng/mL) for 24 hrs prior to treatment with +/- IFNL3 (100 ng/mL) for an additional 24 hrs. RNA was collected and gene count quantitated by NanoString analysis (nCounter Human Immunology v2 Panel). Genes shown were differentially regulated (>2-fold change) in one or more cell lines after doxycycline and/or IFNL3 treatment. Data are shown as log transformed normalized counts. Supplementary Figure S7: IFNAR1 and IFNAR2 RNA expression levels are unchanged among cell lines and conditions. Data were derived from NanoString analysis (described in Figure 8 and Supplementary Figure S6). Supplementary Figure S8: Western blot analysis of pSTAT1 in HEK293T stable lines. HEK293T-EV and FLAG-IFNLR1 isoform lines were +/− dox treated (100 ng/mL) for 24 hrs prior to mock, IFNL3 (100 ng/mL), or IFNA2 (100 ng/mL) stimulation for 1 hr. Protein was collected from cell lysates and assessed by western blot. The larger of the two observed bands is interpreted to reflect pSTAT1 signal.
