# Supplementary material for: Influence of Canonical and Non-Canonical IFNLR1 Isoform Expression on Interferon Lambda Signaling
Source: Viruses. 2023 Feb 25;15(3):632. doi: 10.3390/v15030632 (PMC10052089; doi:10.3390/v15030632)
Supplement: Supplementary file 1 [file viruses-15-00632-s001.zip › viruses-2168574-supplementary.pdf]

Supplemental Figure

IFNLR1 Isoform	UniProt Protein Sequence	Gene Art Nucleotide Sequence	Construct Protein Sequence
IFNLR1<br>Isoform 1<br><br>Q8IU57-1	MAGPERWGPLLLCLLQAAPGRPRLAPP<br>QNVTLLSQNFSVYLTWLPGLGNPQDVTY<br>FVAYQSSPTRRRWREVEECAGTKELLCS<br>MMCLKKQDLYNKFKGRVRTVSPSSKSP<br>WVESEYLDYLFEVEPAPPVLVLTQTEEILS<br>ANATYQLPPCMPPLDLKYEVAFWKEGA<br>GNKTLFPVTPHGQPVQITLQPAASEHHC<br>LSARTIYTFSVPKYSKFSKPTCFLLEVPEAN<br>WAFLVLPSLLILLLVIAAGGVIWKTLMGN<br>PWFQRAKMPRALDFSGHTHPVATFQPS<br>RPESVNDLFLCPQKELTRGVRPTPRVRAP<br>ATQQTRWKKDLAEDEEEEDEEDTEDGV<br>SFQPYIEPPSFLGQEHQAPGHSEAGGVD<br>SGRPRAPLVPSEGSSAWDSSDRSWAST<br>VDSSWDRAGSSGYLAEKGPGQGPGGD<br>GHQESLPPPEFSKDSGFLEELPEDNLSSW<br>ATWGTLPPEPNVPGGPPVSLQTLTFCW<br>ESSPEEEEEARESEIEDSDAGSWGAESTQ<br>RTEDRGRTLGHYMAR	ATGGCTGGACCTGAAAGATGGGGACCTCTGCTGCTGTGCCTGCTGCAAGCTGCTCCTGGCGACTACAAGGACCACGACGGCGATTATA<br>AGGATCACGACATCGACTACAAAGACGATGACGACAAGCGGCCCAGACTGGCCCCTCCTCAGAATGTGACACTGCTGAGCCAGAACTT<br>CAGCGTGTACCTGACATGGCTGCCCGGCCTGGGAAATCCACAGGACGTGACATACTTCGTGGCCTACCAGAGCAGCCCTACCAGACGA<br>AGATGGCGCGAGGTGGAAGAGTGCGCCGGCACAAAAGAACTGCTGTGCAGCATGATGTGCCTGAAGAAGCAGGACCTGTACAACAA<br>GTTCAAGGGCAGAGTGCGGACCGTGTCTCCCAGCTCTAAGTCTCCTTGGGTCGAGAGCGAGTACCTGGACTACCTGTTTGAGGTGGAA<br>CCCGCTCCTCCTGTGCTGGTGCTGACACAGACAGAGGAAATCCTGAGCGCCAACGCCACCTACCAGCTGCCACCTTGTATGCCTCCACT<br>GGATCTGAAGTACGAGGTGGCCTTCTGGAAAGAAGGCGCCGGAAACAAGACCCTGTTTCCTGTGACACCTCACGGCCAGCCTGTGCA<br>GATTACACTGCAGCCTGCCGCCTCTGAGCACCACTGTCTGTCTGCCAGAACCATCTACACCTTTAGCGTGCCCAAGTACTCCAAGTTCAG<br>CAAGCCTACCTGCTTCCTGCTGGAAGTGCCCGAAGCCAATTGGGCCTTTCTGGTGCTGCCTAGCCTGCTGATTCTGCTGCTCGTGATTGC<br>TGCCGGCGGAGTGATCTGGAAAACCCTGATGGGCAACCCCTGGTTCCAGCGGGCCAAAATGCCTAGAGCACTGGACTTCAGCGGCCA<br>CACACATCCCGTGGCCACCTTTCAGCCTAGCAGACCTGAGAGCGTGAACGACCTGTTCCTGTGTCCTCAGAAAGAACTGACCAGAGGC<br>GTGCGGCCCACACCTAGAGTTAGAGCACCAGCCACACAGCAGACCCGGTGGAAGAAAGATCTGGCCGAGGACGAGGAAGAGGAGG<br>ACGAAGAGGATACCGAGGACGGCGTTAGCTTCCAGCCTTATATCGAGCCTCCTAGCTTTCTGGGCCAAGAGCATCAGGCCCCTGGACA<br>CTCTGAAGCTGGCGGAGTTGATAGCGGCAGACCCAGAGCACCTCTGGTGCCAAGCGAAGGATCTAGCGCCTGGGACAGCAGCGATAG<br>AAGCTGGGCCAGCACAGTGGATAGCAGCTGGGATAGAGCCGGCAGCTCTGGATACCTGGCCGAGAAAGGACCTGGTCAAGGACCAG<br>GCGGAGATGGCCACCAAGAATCTCTGCCTCCACCTGAGTTCTCCAAGGACAGCGGCTTCCTGGAAGAACTGCCCGAGGACAATCTGTC<br>CAGCTGGGCTACATGGGGCACACTGCCTCCAGAGCCTAATCTGGTTCCTGGCGGACCTCCTGTGTCTCTGCAGACCCTGACCTTCTGTT<br>GGGAGTCTAGCCCCGAGGAAGAAGAAGAGGCCAGAGAGTCCGAGATCGAGGATTCTGATGCCGGATCTTGGGGAGCCGAGAGCACC<br>CAGAGAACCGAGGATAGAGGCAGAACCCTGGGCCACTACATGGCCAGATAA	MAGPERWGPLLLCLLQAAPGDYKDHDGDYK<br>DHDIDYKDDDDKRPRLAPPQNVTLLSQNFSV<br>YLTWLPGLGNPQDVTYFVAYQSSPTRRRWR<br>EVEECAGTKELLCSMMCLKKQDLYNKFKGRV<br>RTVSPSSKSPWVESEYLDYLFEVEPAPPVLVLT<br>QTEEILSANATYQLPPCMPPLDLKYEVAFWKE<br>GAGNKTLFPVTPHGQPVQITLQPAASEHHCL<br>SARTIYTFSVPKYSKFSKPTCFLLEVPEANWAF<br>LVLPSLLILLLVIAAGGVIWKTLMGNPWFQRA<br>KMPRALDFSGHTHPVATFQPSRPESVNDLFL<br>CPQKELTRGVRPTPRVRAPATQQTRWKKDL<br>AEDEEEEDEEDTEDGVSFQPYIEPPSFLGQEH<br>QAPGHSEAGGVDSGRPRAPLVPSEGSSAWD<br>SSDRSWASTVDSSWDRAGSSGYLAEKGPGQ<br>GPGGDGHQESLPPPEFSKDSGFLEELPEDNLS<br>SWATWGTLPPEPNLVPGGPPVSLQTLTFCW<br>ESSPEEEEEARESEIEDSDAGSWGAESTQRTE<br>DRGRTLGHYMAR
IFNLR1<br>Isoform 2<br><br>Q8IU57-2	MAGPERWGPLLLCLLQAAPGRPRLAPP<br>QNVTLLSQNFSVYLTWLPGLGNPQDVTY<br>FVAYQSSPTRRRWREVEECAGTKELLCS<br>MMCLKKQDLYNKFKGRVRTVSPSSKSP<br>WVESEYLDYLFEVEPAPPVLVLTQTEEILS<br>ANATYQLPPCMPPLDLKYEVAFWKEGA<br>GNKTLFPVTPHGQPVQITLQPAASEHHC<br>LSARTIYTFSVPKYSKFSKPTCFLLEVPEAN<br>WAFLVLPSLLILLLVIAAGGVIWKTLMGN<br>PWFQRAKMPRALELTRGVRPTPRVRAP<br>ATQQTRWKKDLAEDEEEEDEEDTEDGV<br>SFQPYIEPPSFLGQEHQAPGHSEAGGVD<br>SGRPRAPLVPSEGSSAWDSSDRSWAST<br>VDSWDRAGSSGYLAEKGPGQGPGGDG<br>HQESLPPPEFSKDSGFLEELPEDNLSSWA<br>TWGTLPPEPNLVPGGPPVSLQTLTFCWE<br>SSPEEEEEARESEIEDSDAGSWGAESTQR<br>TEDRGRTLGHYMAR	ATGGCTGGACCTGAAAGATGGGGACCTCTGCTGCTGTGCCTGCTGCAAGCTGCTCCTGGCGACTACAAGGACCACGACGGCGATTATA<br>AGGATCACGACATCGACTACAAAGACGATGACGACAAGCGGCCCAGACTGGCCCCTCCTCAGAATGTGACACTGCTGAGCCAGAACTT<br>CAGCGTGTACCTGACATGGCTGCCCGGCCTGGGAAATCCACAGGACGTGACATACTTCGTGGCCTACCAGAGCAGCCCTACCAGACGA<br>AGATGGCGCGAGGTGGAAGAGTGCGCCGGCACAAAAGAACTGCTGTGCAGCATGATGTGCCTGAAGAAGCAGGACCTGTACAACAA<br>GTTCAAGGGCAGAGTGCGGACCGTGTCTCCCAGCTCTAAGTCTCCTTGGGTCGAGAGCGAGTACCTGGACTACCTGTTTGAGGTGGAA<br>CCCGCTCCTCCTGTGCTGGTGCTGACACAGACAGAGGAAATCCTGAGCGCCAACGCCACCTACCAGCTGCCACCTTGTATGCCTCCACT<br>GGATCTGAAGTACGAGGTGGCCTTCTGGAAAGAAGGCGCCGGAAACAAGACCCTGTTTCCTGTGACACCTCACGGCCAGCCTGTGCA<br>GATTACACTGCAGCCTGCCGCCTCTGAGCACCACTGTCTGTCTGCCAGAACCATCTACACCTTTAGCGTGCCCAAGTACTCCAAGTTCAG<br>CAAGCCTACCTGCTTCCTGCTGGAAGTGCCCGAAGCCAATTGGGCCTTTCTGGTGCTGCCTAGCCTGCTGATTCTGCTGCTCGTGATTGC<br>TGCCGGCGGAGTGATCTGGAAAACCCTGATGGGCAACCCCTGGTTCCAGCGGGCCAAAATGCCTAGAGCACTGGAACTGACAAGAGG<br>CGTGCGGCCCACACCTAGAGTTAGAGCACCAGCCACACAGCAGACCCGGTGGAAGAAAGATCTGGCCGAGGACGAGGAAGAGGAGG<br>ACGAAGAGGATACCGAGGACGGCGTGTCCTTCCAGCCTTATATCGAGCCTCCTAGCTTTCTGGGCCAAGAGCATCAGGCCCCTGGACA<br>CTCTGAAGCTGGCGGAGTTGATAGCGGCAGACCCAGAGCACCTCTGGTGCCAAGCGAAGGATCTAGCGCCTGGGACAGCAGCGATAG<br>AAGCTGGGCCAGCACAGTGGATAGCAGCTGGGATAGAGCCGGCAGCTCTGGATACCTGGCCGAGAAAGGACCTGGTCAAGGACCAG<br>GCGGAGATGGCCACCAAGAATCTCTGCCTCCACCTGAGTTCTCCAAGGACAGCGGCTTCCTGGAAGAACTGCCCGAGGACAATCTGTC<br>CAGCTGGGCTACATGGGGCACACTGCCTCCAGAGCCTAATCTGGTTCCTGGCGGACCTCCTGTGTCTCTGCAGACCCTGACCTTCTGTT<br>GGGAGTCTAGCCCCGAGGAAGAAGAAGAGGCCAGAGAGTCCGAGATCGAGGATTCTGATGCCGGATCTTGGGGAGCCGAGAGCACC<br>CAGAGAACCGAGGATAGAGGCAGAACCCTGGGCCACTACATGGCCAGATAA	MAGPERWGPLLLCLLQAAPGDYKDHDGDYK<br>DHDIDYKDDDDKRPRLAPPQNVTLLSQNFSV<br>YLTWLPGLGNPQDVTYFVAYQSSPTRRRWR<br>EVEECAGTKELLCSMMCLKKQDLYNKFKGRV<br>RTVSPSSKSPWVESEYLDYLFEVEPAPPVLVLT<br>QTEEILSANATYQLPPCMPPLDLKYEVAFWKE<br>GAGNKTLFPVTPHGQPVQITLQPAASEHHCL<br>SARTIYTFSVPKYSKFSKPTCFLLEVPEANWAF<br>LVLPSLLILLLVIAAGGVIWKTLMGNPWFQRA<br>KMPRALELTRGVRPTPRVRAPATQQTRWKK<br>DLAEDEEEEDEEDTEDGVSFQPYIEPPSFLGQ<br>EHQAPGHSEAGGVDSGRPRAPLVPSEGSSA<br>WDSSDRSWASTVDSSWDRAGSSGYLAEKGP<br>GQGPGGDGHQESLPPPEFSKDSGFLEELPED<br>NLSSWATWGTLPPEPNLVPGGPPVSLQTLTF<br>CWESSPEEEEEARESEIEDSDAGSWGAESTQ<br>RTEDRGRTLGHYMAR
IFNLR1<br>Isoform 3<br><br>Q8IU57-4	MAGPERWGPLLLCLLQAAPGRPRLAPP<br>QNVTLLSQNFSVYLTWLPGLGNPQDVTY<br>FVAYQSSPTRRRWREVEECAGTKELLCS<br>MMCLKKQDLYNKFKGRVRTVSPSSKSP<br>WVESEYLDYLFEVEPAPPVLVLTQTEEILS<br>ANATYQLPPCMPPLDLKYEVAFWKEGA<br>GNKTLFPVTPHGQPVQITLQPAASEHHC<br>LSARTIYTFSVPKYSKFSKPTCFLLEVPGLF<br>WTHTPCGNLSAQQTRVRE	ATGGCTGGACCTGAAAGATGGGGACCTCTGCTGCTGTGCCTGCTGCAAGCTGCTCCTGGCGACTACAAGGACCACGACGGCGATTATA<br>AGGATCACGACATCGACTACAAAGACGATGACGACAAGCGGCCCAGACTGGCCCCTCCTCAGAATGTGACACTGCTGAGCCAGAACTT<br>CAGCGTGTACCTGACATGGCTGCCCGGCCTGGGAAATCCACAGGACGTGACATACTTCGTGGCCTACCAGAGCAGCCCTACCAGACGA<br>AGATGGCGCGAGGTGGAAGAGTGCGCCGGCACAAAAGAACTGCTGTGCAGCATGATGTGCCTGAAGAAGCAGGACCTGTACAACAA<br>GTTCAAGGGCAGAGTGCGGACCGTGTCTCCCAGCTCTAAGTCTCCTTGGGTCGAGAGCGAGTACCTGGACTACCTGTTTGAGGTGGAA<br>CCCGCTCCTCCTGTGCTGGTGCTGACACAGACAGAGGAAATCCTGAGCGCCAACGCCACCTACCAGCTGCCACCTTGTATGCCTCCACT<br>GGATCTGAAGTACGAGGTGGCCTTCTGGAAAGAAGGCGCCGGAAACAAGACCCTGTTTCCTGTGACACCTCACGGCCAGCCTGTGCA<br>GATTACACTGCAGCCTGCCGCCTCTGAGCACCACTGTCTGTCTGCCAGAACCATCTACACCTTTAGCGTGCCCAAGTACTCCAAGTTCAG<br>CAAGCCTACCTGCTTCCTGCTGGAAGTGCCTGGCCTGTTCTGGACCCACACACCTTGTGGAAACCTGTCTGCCCAGCAGACAAGAGTGC<br>GGGAATGA	MAGPERWGPLLLCLLQAAPGDYKDHDGDYK<br>DHDIDYKDDDDKRPRLAPPQNVTLLSQNFSV<br>YLTWLPGLGNPQDVTYFVAYQSSPTRRRWR<br>EVEECAGTKELLCSMMCLKKQDLYNKFKGRV<br>RTVSPSSKSPWVESEYLDYLFEVEPAPPVLVLT<br>QTEEILSANATYQLPPCMPPLDLKYEVAFWKE<br>GAGNKTLFPVTPHGQPVQITLQPAASEHHCL<br>SARTIYTFSVPKYSKFSKPTCFLLEVPGLFWTH<br>TPCGNLSAQQTRVRE

Supplemental Figure S1: Protein and nucleotide sequences of IFNLR1 isoforms. Unaltered protein sequences downloaded from UniProt database (**left** column), codon-optimized nucleotide sequences used for Gene Art synthesis (**middle** column), and FLAG-tagged IFNLR1 isoform protein sequences (**right** column). Signal sequence is denoted in blue; 3x-FLAG tag is denoted in red; the transmembrane domain is denoted in orange.

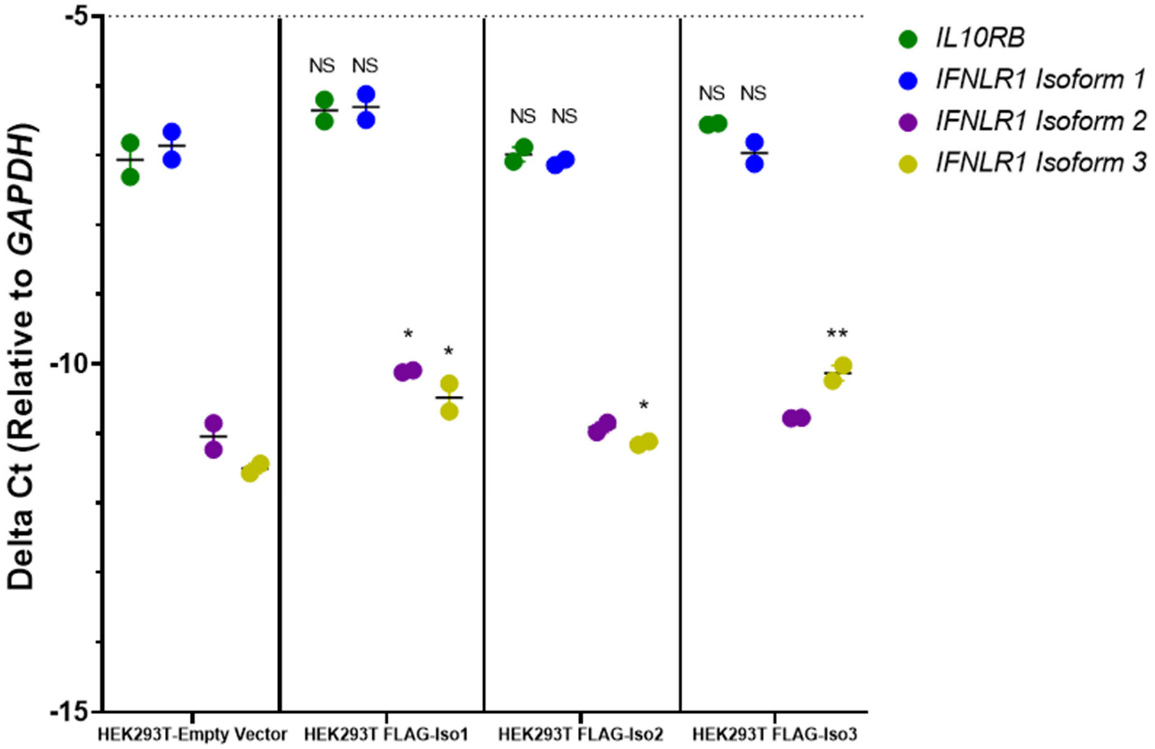

Supplemental Figure S2: Endogenous *IL10RB* and *IFNLR1* isoform expression in HEK293T stable lines. Cellular gene expression was quantitated by qRT-PCR. Statistical significance represented by asterisks reflect comparisons between EV and FLAG-IFNLR1 isoform cells. Error bars represent standard error of the mean. * = $p < 0.05$, ** = $p < 0.01$. NS = not significant.

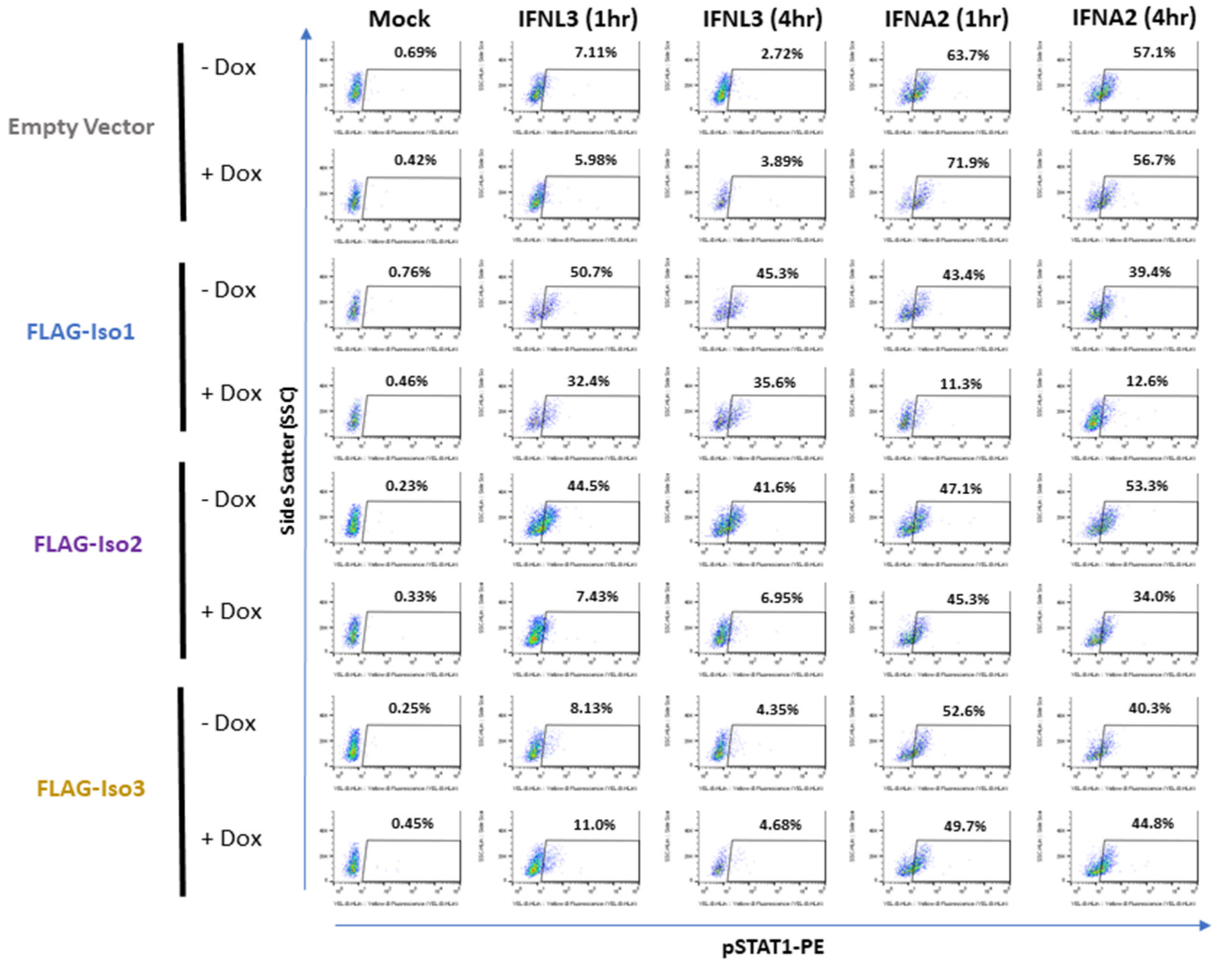

Supplemental Figure S3: Representative flow cytometry plots used for quantification in Figures 3, 4, 6, and 9

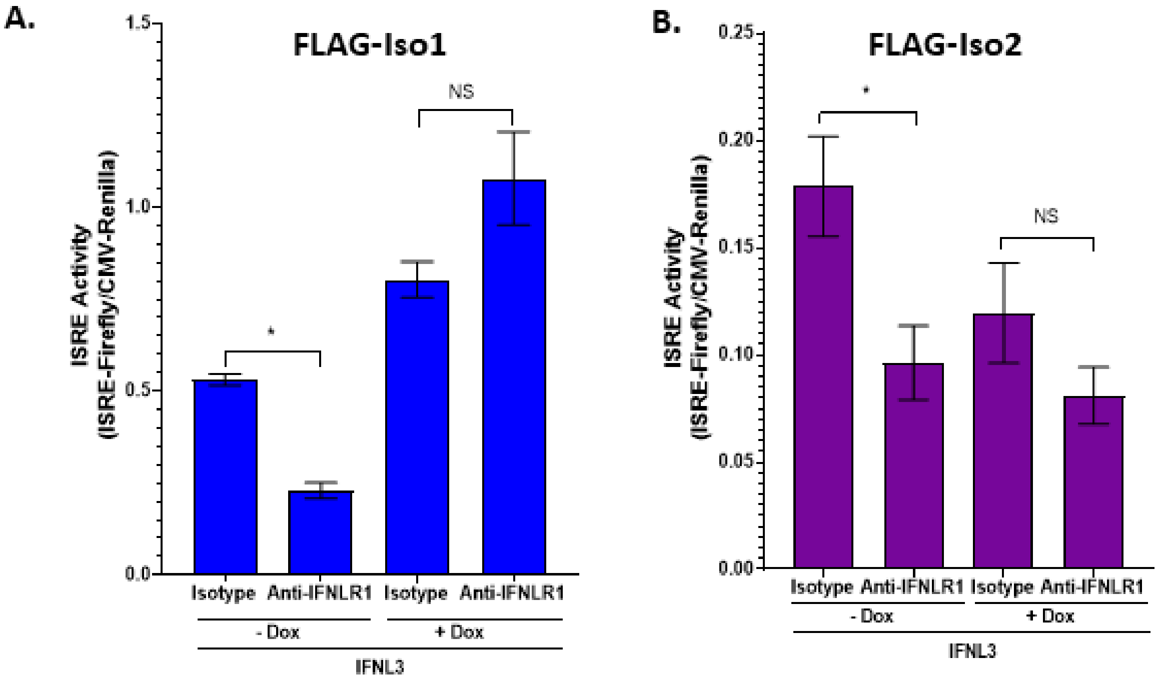

Supplemental Figure S4: Effect of pre-incubation with anti-IFNLR1 antibody on the IFNL3 response in FLAG-Iso1 and FLAG-Iso2 lines. IFNLR1 FLAG-Iso1 **(A)** and FLAG-Iso2 **(B)** cells were treated +/- dox (100 ng/ml) for 24 hrs prior to incubation with anti-IFNLR1 antibody or isotype control (anti-IL-28RA antibody, control IgG, R&D Systems, 4ug/ml) for 1.5 hrs, after which the cells were stimulated with IFNL3 (100 ng/ml) for 24 hrs and harvested for dual luciferase assay (representative data, 2 independent experiments). Error bars represent standard error of the mean. * = $p < 0.05$. NS = not significant.

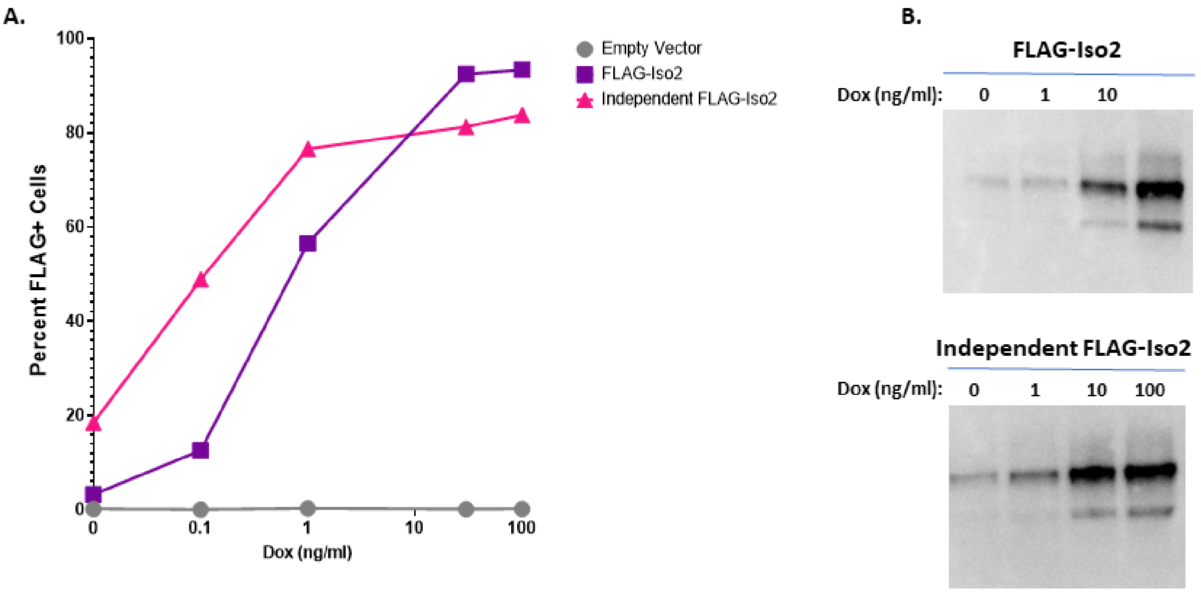

Supplemental Figure S5: Dox-titratable FLAG-Isoform 2 expression in independent HEK293T stable lines. (A) HEK293T-EV, the FLAG-Iso2 line characterized in Figure 4, and an independent FLAG-Iso2 line were treated +/- dox (0, 0.1, 1, 10, or 100 ng/ml) for 24 hr prior to analysis of FLAG expression by flow cytometry. **(B)** FLAG-Iso2 expression in each line after treatment +/- dox (0, 1, 10, 100 ng/ml) for 24 hr is shown by western blot of cell lysates using anti-FLAG antibody.

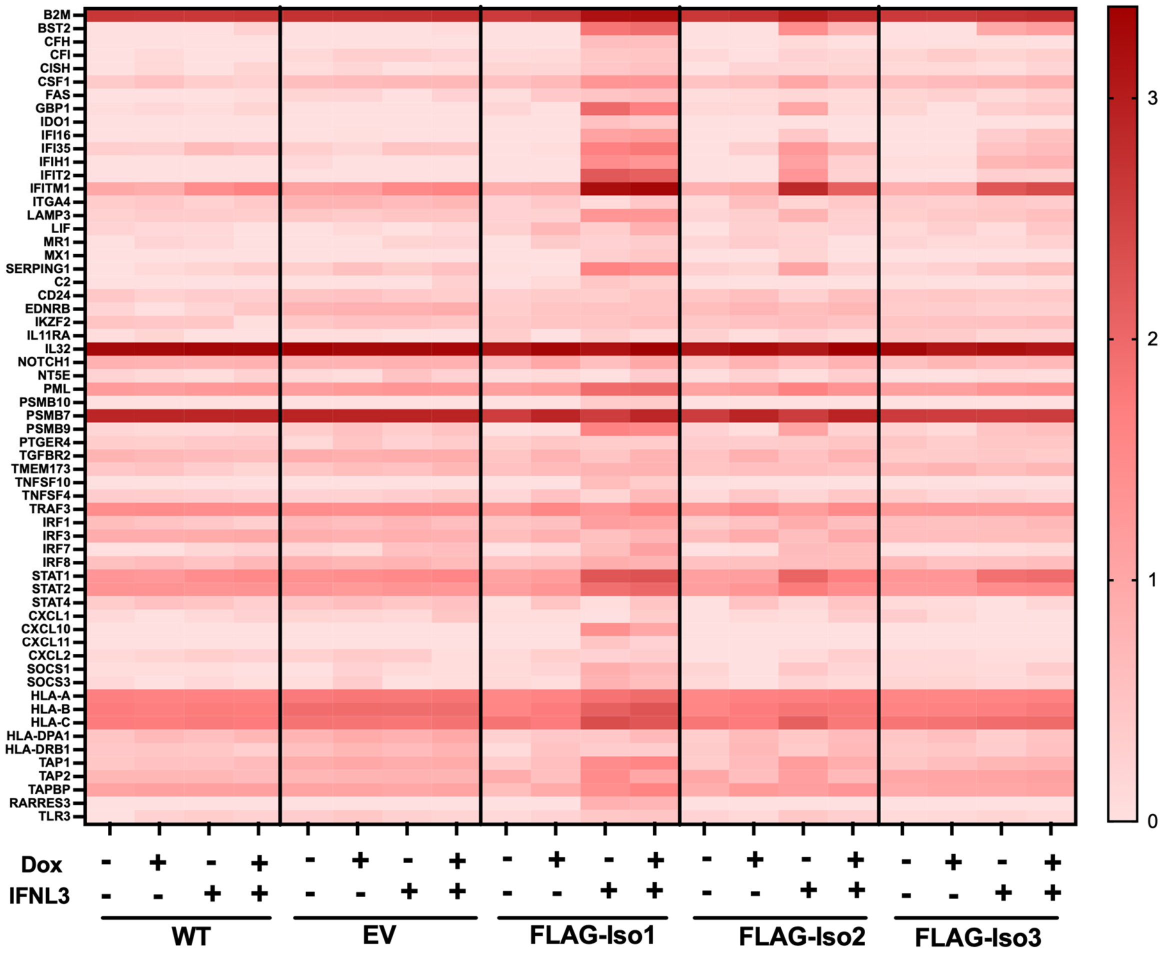

Supplementary Figure S6: Differentially expressed genes among HEK293T cells line. HEK293T WT and stable lines were induced +/- dox (100 ng/ml) for 24 hrs prior to treatment with +/- IFNL3 (100 ng/ml) for an additional 24 hrs. RNA was collected and gene count quantitated by NanoString analysis (nCounter Human Immunology v2 Panel). Genes shown were differentially regulated (>2-fold change) in one or more cell lines after doxycycline and/or IFNL3 treatment. Data are shown as log transformed normalized counts.

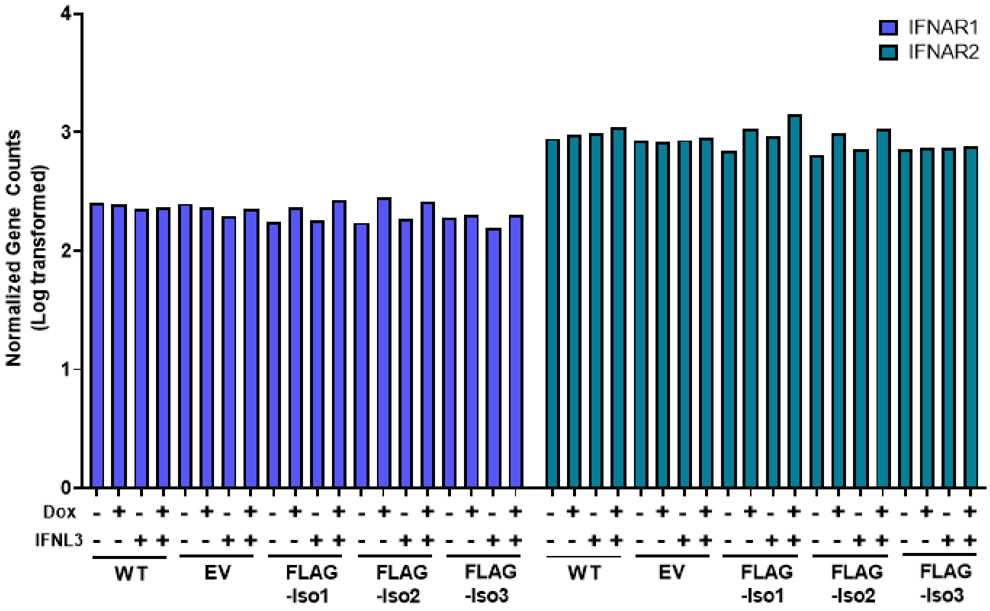

Supplemental Figure S7: ***IFNAR1*** **and** ***IFNAR2*** **RNA expression levels are unchanged among cell lines and conditions.** Data were derived from NanoString analysis (described in Figure 8 and Supplemental Figure S6).

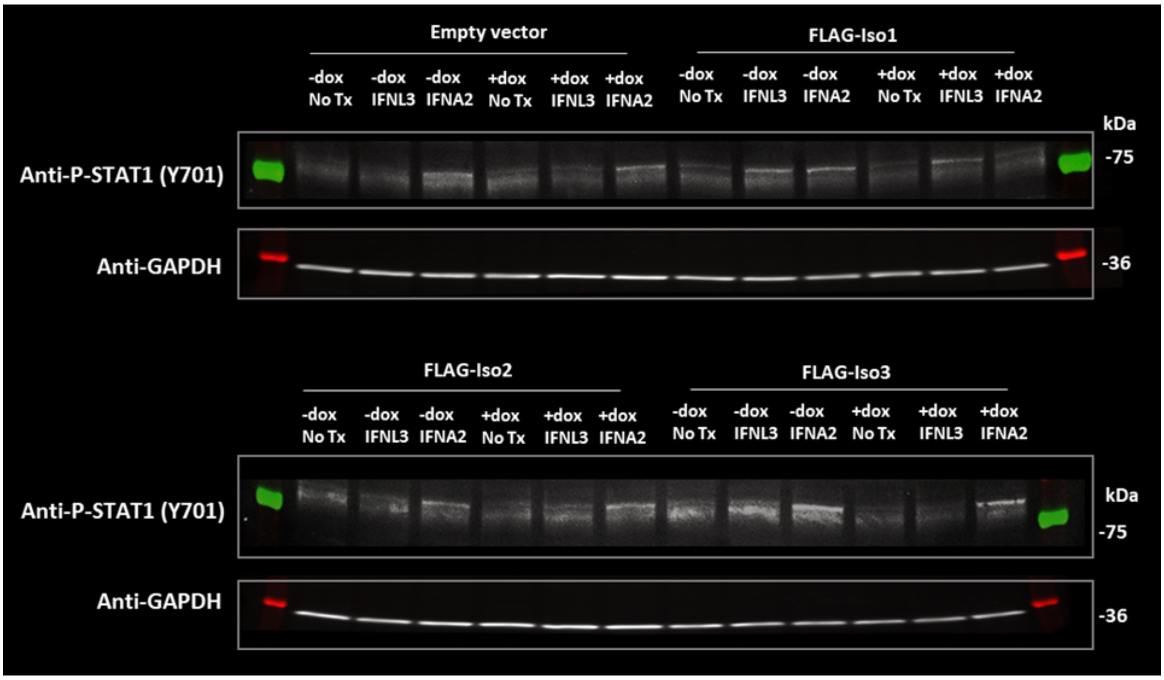

Supplemental Figure S8: Western blot analysis of pSTAT1 in HEK293T stable lines. HEK293T-EV and FLAG-IFNLR1 isoform lines were +/- dox treated (100 ng/ml) for 24 hrs prior to mock, IFNL3 (100ng/ml), or IFNA2 (100 ng/ml) stimulation for 1 hr. Protein was collected from cell lysates and assessed by western blot. The larger of the two observed bands is interpreted to reflect pSTAT1 signal.
